# The effects of ketamine on dopaminergic function: meta-analysis and review of the implications for neuropsychiatric disorders

**DOI:** 10.1038/mp.2017.190

**Published:** 2017-10-03

**Authors:** M Kokkinou, A H Ashok, O D Howes

**Affiliations:** 1 Robert Steiner MR Unit, Psychiatric Imaging Group, MRC London Institute of Medical Sciences (LMS), Hammersmith Hospital, London, UK; 2 Psychiatric Imaging Group, Faculty of Medicine, MRC London Institute of Medical Sciences (LMS), Imperial College London, London, UK; 3 Department of Psychosis Studies, Institute of Psychiatry, Psychology & Neuroscience, Kings College London, London, UK

## Abstract

Ketamine is a non-competitive antagonist at the *N*-methyl-d-aspartate receptor. It has recently been found to have antidepressant effects and is a drug of abuse, suggesting it may have dopaminergic effects. To examine the effect of ketamine on the dopamine systems, we carried out a systematic review and meta-analysis of dopamine measures in the rodent, human and primate brain following acute and chronic ketamine administration relative to a drug-free baseline or control condition. Systematic search of PubMed and PsychInfo electronic databases yielded 40 original peer-reviewed studies. There were sufficient rodent studies of the acute effects of ketamine at sub-anaesthetic doses for meta-analysis. Acute ketamine administration in rodents is associated with significantly increased dopamine levels in the cortex (Hedge’s *g*= 1.33, *P<0.01*), striatum (Hedge’s *g*=0.57, *P<0.05*) and the nucleus accumbens (Hedge’s *g*=1.30, *P*<*0.05*) compared to control conditions, and 62–180% increases in dopamine neuron population activity. Sub-analysis indicated elevations were more marked in *in vivo* (*g*=1.93) than *ex vivo* (*g*=0.50) studies. There were not enough studies for meta-analysis in other brain regions studied (hippocampus, ventral pallidum and cerebellum), or of the effects of chronic ketamine administration, although consistent increases in cortical dopamine levels (from 88 to 180%) were reported in the latter studies. In contrast, no study showed an effect of anaesthetic doses (>100 mg kg^−1^) of ketamine on dopamine levels *ex vivo*, although this remains to be tested *in vivo*. Findings in non-human primates and in human studies using positron emission tomography were not consistent. The studies reviewed here provide evidence that acute ketamine administration leads to dopamine release in the rodent brain. We discuss the inter-species variation in the ketamine induced dopamine release as well as the implications for understanding psychiatric disorders, in particular substance abuse, schizophrenia, and the potential antidepressant properties of ketamine, and comparisons with stimulants and other NMDA antagonists. Finally we identify future research needs.

## Introduction

Ketamine, a phencyclidine (PCP) hydrochloride derivative, is used as a common dissociative anaesthetic and in pain treatment. It is a non-competitive antagonist at the *N*-methyl-d-aspartate (NMDA) excitatory ligand-gated ion channel and binds to the PCP-binding site of the receptor to prevent the influx of Ca^+2^ ions following binding by glycine and glutamate co-agonists.^1^

Ketamine is a ‘club drug’ of abuse. The annual prevalence of ketamine use in young adults ranges from 0.8 to 1.8%.^2^ At sub-anaesthetic doses ketamine induces behavioural and neurochemical alterations associated with symptoms of schizophrenia in humans.^3, 4, 5^ Interestingly, ketamine has recently emerged as a potential treatment for major depressive disorder.^6^ A single dose of 0.5 mg kg^−1^ of ketamine has been shown to have a rapid and relatively potent antidepressant effects.^7, 8^ However the dose of ketamine used in the treatment of resistant major depression is similar to that shown to have psychotomimetic and cognitive effects.^9^

The abuse potential and psychotomimetic effects of ketamine have been linked to the dopaminergic system. Moreover, its dopaminergic effects may also contribute to its antidepressant effects. In view of this we aimed to systematically review and meta-analyse the evidence that sub-anaesthetic doses of ketamine affect dopamine levels. In addition we summarise findings at anaesthetic doses to enable comparison.

## Methods

### Search strategy and study selection

A PubMed and Psych INFO electronic database search was performed using the search terms “ketamine” AND “dopamine” from July 1972 to mid-July 2016 (see Supplementary Figure 1 for study selection details). Inclusion criteria were: (1) racemic ketamine administered at sub-anaesthetic doses (⩽100 mg kg^−1^ i.p. in rodents), (2) measures of dopamine levels in brain. Exclusion criteria were (1) studies which used ketamine at anaesthetic doses (>100 mg kg^−1^ i.p. in rodents), (2) studies which lacked a baseline condition or control group, (3) studies that were in non-English language, (4) studies that did not report original data, (5) not reporting the s.d.’s or s.e.m, (6) *in vitro* studies, and (7) studies that did not report dopamine levels although they may report other dopaminergic outcome measures (for example, metabolite levels). We used the same search criteria to identify non-human primate and human studies but did not apply a dose cut-off as this may vary by species and route of administration. We used 100 mg kg^−1^ as the upper dose limit in rodents as ketamine is used at greater than this as a starting dose for injectable anaesthesia in rodents and we aimed to focus on sub-anaesthetic doses associated with behavioural and psychological effects.^10^ However, to enable comparison with the effects of ketamine at anaesthetic doses, we also performed a separate search using the search terms ‘ketamine’ AND (‘high dose’ OR ‘anaesthesia’ OR ‘anaesthetic’) AND ‘dopamine’ and summarise the results of studies investigating the effect of anaesthetic doses of ketamine administration on dopaminergic outcomes relative to control condition.

### Data extraction and statistical analyses

The following variables were extracted from all the studies: authors, year of publication, subject characteristics of the control and ketamine group (species, strain, sex, age and weight), dopaminergic measure characteristics (method, duration of ketamine treatment, the dose of ketamine used, route of administration of the drug, the dopaminergic outcome measure under investigation, and time the outcome was investigated in relation to the injection of the drug).

The main outcome measure was the effect size for the difference in dopamine levels following ketamine administration relative to a drug-free baseline or control condition. Where a mean and variance was not reported in the text, data were extracted from graphs and percentage increase and decrease were calculated. Fold change was converted to a percentage value for consistency. Plot digitizer software was used to examine reliability for the data from studies where data were available only in a plot format (http://plotdigitizer.sourceforge.net/). When data are provided for more than one time point, the time point which showed the largest percentage change was chosen for all studies for consistency.

A meta-analysis and sub-analyses for specific methods (that is, measurement of dopamine level by (1) *in vivo*= microdialysis and fast-scan voltammetry; or (2) *ex vivo*= following decapitation) were performed when there were at least five studies investigating dopamine levels in a specific region of interest. Where studies investigated dopamine effects following two or more doses of ketamine administration, the dose which documented the highest percentage change was used for the meta-analysis. For these reasons the effect sizes in the meta-analyses should be considered as the largest potential effect of ketamine on the dopamine measures.

The statistical analyses of the extracted data were conducted using the R statistical programming language version 3.2.2 with the ‘metafor’ package. The main outcome measure was the effect size for the dopaminergic index in the cortex, striatum and nucleus accumbens following acute ketamine administration using a random effects model. Publication bias was assessed using funnel plots as well as regression tests. Heterogeneity was estimated using the *I*^2^ value (*I*^2^ values <50% indicate low to moderate heterogeneity, whereas *I*^2^ >50% indicate moderate to high heterogeneity). Leave-one-out sensitivity analyses were conducted. A significance level of *P*<0.05 (two-tailed) was taken as significant. Meta-analysis was conducted where, there were more than 5 studies in a brain area as results with <5 studies might be unstable. In regions where there were not enough data for a meta-analysis, we have summarised the findings in the table. Publication bias and sensitivity analyses were conducted for meta-analyses including at least 8 original studies.

### Study sample and methodological characteristics

The literature search identified 1263 potentially relevant articles for initial screening. Duplications (*N*=424) were identified using a function in Endnote and confirmed by manual screening of the titles. We excluded 774 studies from first assessment of titles and abstracts. Sixty-five abstracts were classified as possible for inclusion and full texts were obtained. Forty papers were excluded from further analysis. Of the 25 included studies, 21 investigated changes in dopaminergic measure following ketamine treatment in the rodent brain and four in the primate brain. A total of fifteen studies were included in meta-analyses (see Supplementary Figure 1 for a PRISMA diagram of literature search).

Supplementary Table 1 lists the subject characteristics for the included studies identified from our main and separate searches. From the studies reporting animal sex, all studies were done in male animals. Twenty rodent studies investigated the acute effects of ketamine, whereas only six studies investigated the chronic effects (four of which investigated both acute and chronic effects of ketamine on dopaminergic systems; Supplementary Tables 2 and 4). Out of the four primate studies, two used a constant infusion of ketamine while the other two studies investigated the effects of a single ketamine injection (Supplementary Table 6).

## Results

### Meta-analysis of dopamine levels in the frontal cortex following acute ketamine administration in rodents

**Figure 1:**
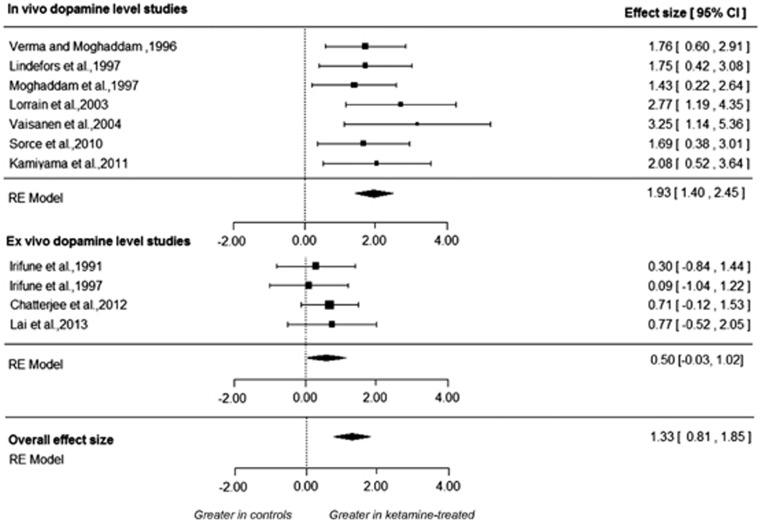
The effect of acute ketamine on frontal cortical dopamine levels. Meta analysis of dopamine levels in the frontal cortex following acute ketamine administration. There was a large significant overall effect of ketamine on dopamine measures (summary effect size=1.33, p<0.001). Sub-analysis pooled effect size shown for *in vivo* microdialysis studies in the top panel, *ex vivo* dopamine level studies in the bottom panel and overall effect size for both microdialysis and *ex vivo* studies combined.

Meta-analysis of 11 studies involving 72 ketamine-treated and 70 vehicle-treated rodents, showed a significant increase in dopamine levels in the cortex after ketamine administration (range of ketamine dose 18–100 mg kg^−1^) compared to control state with an effect size of 1.33 ((95% confidence interval (CI), 0.81–1.85), *P*<0.001; Figure 1).^11, 12, 13, 14, 15, 16, 17, 18, 19, 20, 21^ The sub-analysis of the *in vivo* studies showed a significant increase in dopamine levels in the cortex with an effect size of 1.93 ((95% CI 1.40, 2.45), *P*<0.001). The sub-analysis of the *ex vivo* dopamine level studies did not show a difference in dopamine levels in the ketamine relative to the control condition (effect size=0.50 (CI,−0.03–1.02), *P*=0.064).

There was evidence of significant heterogeneity among the studies (*I*^2^=45.8% (95% CI, 0–85.54%); *P*<0.05). The regression test for funnel plot asymmetry was significant (*t*=3.31, df=9 and *P*=0.01), suggesting publication bias is likely. Trim-fill analysis estimated three missing studies on the left side. The results remained significant after correcting for putatively missing studies (effect size: 1.02; CI, 0.46–1.59, *P*<0.01; Supplementary Figure 2). The summary effect size reached significance in all cases in the leave-one-out analysis, with summary effect sizes varying from 1.19 to 1.45 (all *P*<0.001). A study investigating change in dopaminergic levels following administration of a specific enantiomer of ketamine rather than a racemic mixture of ketamine was excluded from the meta-analysis, but the results of the study were in line with the racemic findings.^22^

### Meta-analysis of dopamine levels in striatum following acute ketamine administration

**Figure 2:**
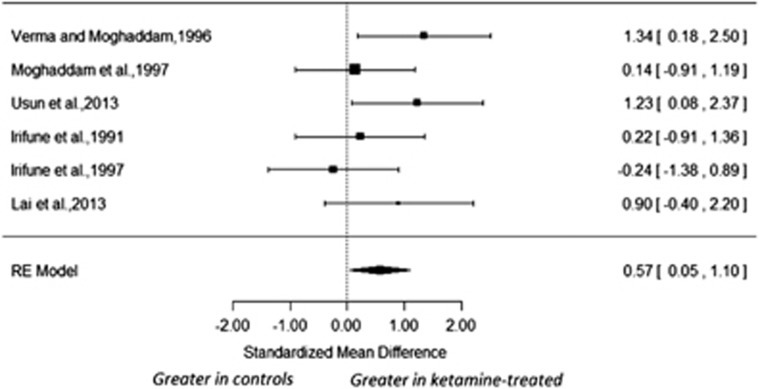
Meta-analysis showing the effect of acute ketamine on striatal dopamine levels. There was a significant increase in dopamine measures following ketamine (effect size=0.57; *P*=0.03).

Meta-analysis of six studies involving 38 ketamine-treated and 38 vehicle-treated rodents, showed a significant increase in dopamine levels following acute ketamine (range of dose 10–50 mg kg^−1^) compared to the control condition (effect size: 0.57; (CI, 0.05–1.10); *P*=0.03; Figure 2).^12, 13, 15, 18, 21, 23^ The *I*^2^ value was 19.96% (95% CI, 0.00–86.38%), indicating low to moderate heterogeneity.

### Meta-analysis of dopamine levels in the nucleus accumbens following acute ketamine administration

**Figure 3:**
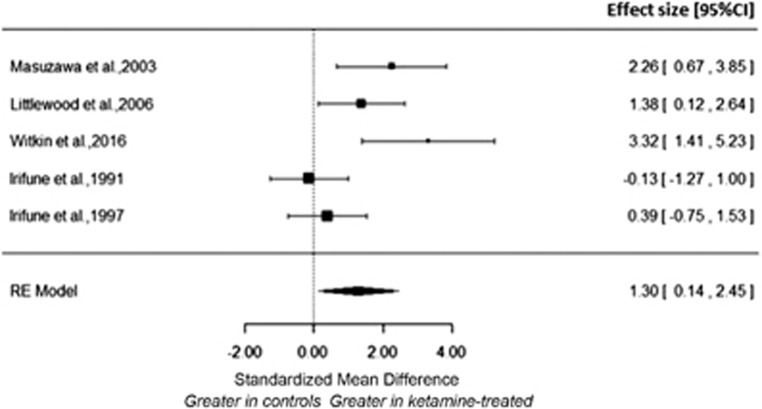
Meta-analysis of the effect of acute ketamine on nucleus accumbens dopamine levels. There was a significant increase in dopamine levels in the nucleus accumbens following ketamine with a large effect size (summary effect size=1.30; *P*=0.028).

Meta-analysis of five studies involving 28 ketamine-treated and 28 vehicle-treated rodents, showed a significant increase in dopamine levels in the ketamine (range of dose 10–100 mg kg^−1^) relative to the control condition with an effect size of 1.30 ((CI, 0.14–2.45), *P*=0.028) Figure 3).^12, 13, 22, 24, 25^ The *I*^2^ value was 79.29% (95% CI, 18.46–97.03%), indicating high heterogeneity.

### Dopamine levels in other brain regions in rodents

There were too few studies to permit meta-analysis in other brain regions. The majority of studies report no changes in dopamine levels in the hippocampus, brainstem and ventral pallidum following acute ketamine administration (Supplementary Table 2).^12, 13, 25^ Likewise, the studies retrieved report no changes in dopamine levels in the hippocampus, midbrain and cerebellum following chronic ketamine administration (Supplementary Table 4).^11, 26^

### Dopamine neuron firing in ventral tegmental area

Four studies assessed dopamine neuron firing in the ventral tegmental area (VTA) of rats following ketamine administration.^22, 27, 28, 29^ Acute ketamine (in three studies) caused an increase in population activity of 62–180%, with no change in firing rate or burst activity in three out of four studies whereas chronic ketamine (1 study) had no significant effect on dopamine neuron firing (Supplementary Table 3).

### Dopamine levels following chronic ketamine administration in rodents

There were too few studies to permit meta-analysis of the effect of chronic ketamine treatment on dopamine levels in any brain region. In total three studies investigated the chronic effect of ketamine administration (range of dose: 15–100 mg kg^−1^ per day) on the percentage difference in dopamine levels in the cortex in rodents for a range of 8 days to 3 months.^11, 16, 30^ All three studies reported increases in dopamine levels in the cortex ranging from 88% to over 180%. In the striatum the results are inconsistent with one out of three studies showing increase in dopamine levels, whilst the other two found no significant effect of ketamine on dopamine levels. A potential explanation of the lack of effect in one study is the relatively low dose and route of administration of ketamine, which was 15 mg kg^−1^ in liquid diet^31^ and the methodology used to assess dopamine levels in the other study^30^ (Supplementary Table 4).

### Effect of anaesthetic doses of ketamine on dopamine levels in rodents

Four studies assessed dopamine levels in cortex, striatum, nucleus accumbens, brainstem and hippocampus of rodents following anaesthetic dose of ketamine administration (range 150–350 mg kg^−1^)).^12, 13, 32, 33^ All four studies consistently reported no change in *ex vivo* dopamine levels (Supplementary Table 5).

### Effects of acute and chronic ketamine on the dopaminergic system in non-human primates and humans

#### Microdialysis studies

A majority of the non-human primate studies (three out of four) showed no effect of ketamine on dopamine levels in the cortex or striatal regions (Supplementary Table 6).^34, 35, 36^ One study showed a small but significant 30% increase in dopamine in the striatum compared to baseline levels following a single acute ketamine injection.^37^ No study investigated the effect of chronic ketamine administration on dopamine levels.

#### Positron emission tomography studies

Table 1 summarises studies on effect of ketamine on the dopaminergic system in non-human primates. The majority of the studies in non-human primates investigated effects of anaesthetic doses (3–10 mg kg^−1^) of ketamine. The one study to investigate dopamine synthesis capacity found ketamine increased dopamine synthesis, although it used an anaesthetic dose.^36^ Interestingly, both anaesthetic^36^ and sub-anaesthetic doses^38^ showed reductions in D2/3 receptor availability, consistent with dopamine release following ketamine. However, findings of the effects of ketamine on dopamine transporter (DAT) availability are variable with sub-anaesthetic doses showing no significant change,^34^ whilst studies using ketamine doses in an anaesthetic range^36, 39, 40^ showed increases in DAT availability. All studies were done in small samples, 3–5 non-human primates, thus findings should be interpreted with caution and more studies with sub-anaesthetic doses of ketamine are needed.

### Effects of acute and chronic ketamine administration on the dopaminergic system in humans

Table 2 summarises the studies of the effects of ketamine challenge on dopamine release in humans. We did not find any studies which investigated the effects of ketamine on dopamine synthesis or transporter levels in human. All studies measured dopamine release following ketamine challenge using positron emission tomography (PET) imaging. Three studies reported evidence of dopamine release, as indexed by a ~14% change in D2/3 radiotracer binding, in the striatum following ketamine administration in healthy volunteers.^41, 42, 43^ Similar results were observed in the cingulate cortex but not in the thalamus or the frontal, temporal and parietal cortices.^44^ However, three studies did not detect dopamine release following ketamine infusion,^45, 46, 47^ although one of these showed that ketamine augmented amphetamine-induced dopamine release.^46^ Methodological factors, for example, the radiotracer imaging not being conducted under equilibrium conditions, may account for the discrepant finding (see refs 48 and 49 for a further discussion of these factors).

However, it should also be noted that striatal dopamine release with ketamine administration ranged from 30 to 60% compared to baseline while cortical regions display 150–250% changes (Supplementary Table 2). To put this in perspective, microdialysis studies in rodents show that amphetamine administration increases striatal dopamine levels to 300–400% compared to baseline,^50^ and this is readily detectable by PET (see review^51^). It has been estimated that the ratio of dopamine release to change in radiotracer binding is about 44:1.^52, 53^ Thus, given the relatively modest degree of dopamine release in the striatum with ketamine, this may be close to the limit of detection with PET techniques.^49^ Recent studies have shown that the agonist ligand [11C] PHNO is more sensitive to quantify amphetamine-induced dopamine release.^54^ Thus future studies with agonist ligands such as [11C] PHNO may clarify this issue (Box 1).

One study in chronic ketamine users reported an upregulation of D1 receptor availability in the frontal cortex.^55^ However, there are no studies investigating the effects of chronic ketamine use on dopamine synthesis, transporter availability or release. Future research should investigate the effect of ketamine on these aspects of dopamine function in humans (Box 1).

## Discussion

**Figure 4:**
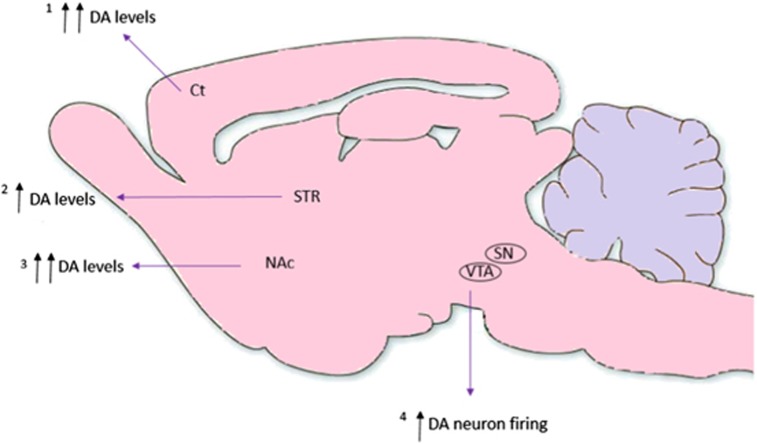
Showing the location of the major dopaminergic findings following acute ketamine administration from our meta-analyses and qualitative review. _1_Meta-analysis finding with effect size of 1.33 [95% CI, 0.81-1.85 p<0.001]. _2_Meta-analysis finding with effect size of 0.57 [95% CI, 0.05 – 1.10 p< 0.05]. _3_Meta-analysis finding with effect size of 1.30 [95% CI, 0.14 – 2.45 p< 0.05]. _4_Found in the acute studies to measure this to date (Supplementary Table 3). Arrows denote the relative increases in dopamine levels in each region of interest.

Our meta-analysis shows that acute ketamine administration increases dopamine levels in the striatum, the nucleus accumbens and the frontal cortex in rodents compared to controls with medium to very large effect sizes (Hedge’s *g*: 0.57, 1.3 and 1.33 respectively). These findings are summarised in Figure 4. Specifically there was evidence for increased dopamine levels following ketamine in the frontal cortex in the majority (9 out of 11) of studies in rodents, with increases ranging from 50 to 400% (Supplementary Table 2). All three studies of the effects of acute ketamine administration on dopamine neuron firing in the VTA of rodents consistently showed an increase in firing (Supplementary Table 3).^22, 27, 28^ Interestingly the effect size of the dopaminergic increase following acute ketamine treatment is numerically higher in the nucleus accumbens and the frontal cortex compared to the striatum. This could be potentially attributed to the higher doses of ketamine used in the studies of these two regions (frontal cortex range of doses: 18–100 mg kg^−1^; nucleus accumbens range of doses: 10–100 mg kg^−1^) compared to the striatum (10–50 mg kg^−1^). Alternatively it could suggest that ketamine preferentially increases dopamine release in the nucleus accumbens and cortex relative to the striatum. Studies directly comparing dopamine release across regions at the same dose of ketamine are needed to test this hypothesis. Although there were too few studies for meta-analysis, we also found evidence for consistent elevation of dopamine levels following chronic ketamine administration in the frontal cortex.^11, 16, 30^ These findings extend evidence of increased dopamine metabolite levels such as Homovanillic acid (HVA), 3,4-dihydroxyphenylacetic acid (DOPAC) and 3-methoxytyramine (3-MT), following acute^11, 13, 16, 56^ and chronic ketamine administration in the cortex.^11, 16, 30^ Taken together these findings indicate that acute ketamine elicits a significant dopaminergic response in cortex, striatum and nucleus accumbens in rodents and suggest that this is also the case with chronic administration (Supplementary Table 4). Nevertheless further *in vivo* studies are needed to determine the effects of chronic ketamine administration on dopamine release (see Box 1 for suggested future directions).

### General methodological considerations

It is important to note that we found heterogeneity across the analyses in the cortex and nucleus accumbens. Our sub-analysis by method for the cortex showed that the *ex vivo* studies, which measured total dopamine content in homogenised tissue, showed no significant effects of ketamine, unlike the *in vivo* studies, where consistent and large elevations were seen. Moreover, where significant differences were detected in *ex vivo* studies, they were more modest than those reported by *in vivo* studies (Table 2), suggesting that *ex vivo* methods may be less sensitive than *in vivo* methods. There were too few studies for separate sub-analyses in the striatum and in the nucleus accumbens but the same pattern of results is seen for *ex vivo* and *in vivo* studies in these regions. Thus, whilst variations between studies in terms of the range of doses of ketamine and the time lapse between the last ketamine treatment may contribute, differences between *ex vivo* and *in vivo* studies are likely to be a major contributor to heterogeneity. However, this variability might be expected to weaken effects rather than explain the elevations we report. Moreover we employed a random effects model, which is robust to heterogeneity in effects. Of note where more than one dose of ketamine was used in a study, the dose which elicited the highest difference in the dopaminergic measure was chosen for the meta-analysis. Thus the effect sizes calculated from these studies should be considered an estimate of the largest likely effect size. In addition we summarised the effects of anaesthetic dose of ketamine on dopamine levels in the rodent brain. All studies showed consistently no change in dopamine levels following anaesthetic dose of ketamine (Supplementary Table 5). However all studies used *ex vivo* methods of dopamine level measurement. This observation highlights the fundamental issue raised by our sub-analysis by method and further supports that *in vivo* studies are required to delineate the anaesthetic ketamine effect on dopaminergic function over the limitations of *ex vivo* methods. Moreover further studies are required to investigate the dose at which the stimulatory effects of ketamine on dopamine function decline. Finally general limitations of included studies are that only male animals were used and there was no report of ketamine brain or plasma levels. Whilst there is no sex difference in the ketamine brain levels in mice and rats,^57, 58^ it has been shown that there are higher numbers of dopaminergic cells in female than male rats.^59^ Thus extrapolations of dopaminergic modulation elicited by ketamine in females should be treated with care and studies in females are needed (Box 1). In addition strain-specific effect of acute and repeated ketamine on dopaminergic function remains to be directly tested (Box 1).

### Mechanism of ketamine’s action on the dopamine system

**Figure 5:**
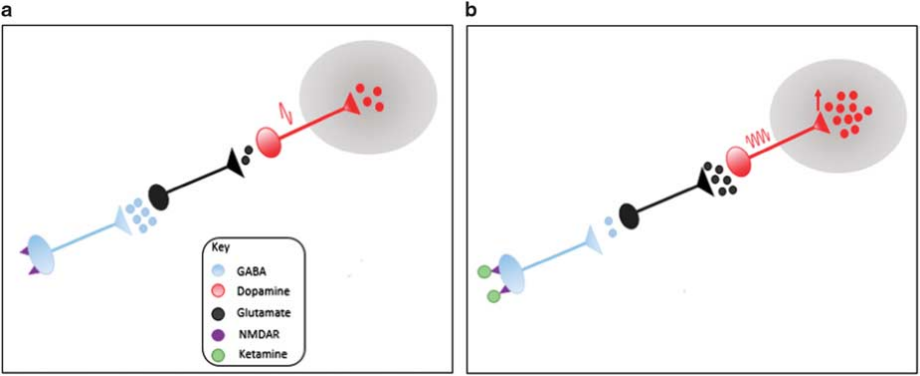
Theorised mechanism of action of ketamine in mediating dopaminergic change. Ketamine (green) blocks NMDA receptors (purple) on GABAergic interneurons (blue) disinhibiting glutamate neurons (black) projecting to dopamine neurons in the midbrain, increasing glutamate release and subsequently increasing dopamine neuron firing (red) and thus increasing dopamine levels in projection targets such as the striatum and cortex.

The mechanism underlying ketamine’s action on dopaminergic neurons remains to be fully established. However, several lines of evidence indicate that this involves *N*-methyl-d-aspartate receptor (NMDAR) blockade on GABAergic interneurons that regulate excitatory projections to the midbrain dopamine neuron cell bodies. Specifically NMDAR antagonists were shown to decrease GABAergic interneuron function^60^ and this in turn leads to an increase in pyramidal cell firing which is thought to lead to increased excitation of dopamine neurons.^61^ In line with this, there is evidence that NMDAR antagonists induce excessive glutamate release.^62, 63, 64, 65^ All three studies of the effects of acute ketamine administration on dopamine neurons in the VTA showed an increase in firing (Supplementary Table 3).^22, 27, 28^ Taken with our meta-analytic findings, this is consistent with the hypothesis that the disinhibition of glutamatergic projections onto dopamine neurons increases activation of dopaminergic neurons (Figure 5), although it remains to be directly shown that this is solely due to glutamate release.

### Comparison with stimulants

In the rodent brain the magnitude of dopamine changes is lower with ketamine relative to those seen with amphetamine and cocaine. Unlike ketamine, amphetamine at doses of 0.25 mg kg^−1^ and 1 mg kg^−1^ increased dopamine output with a maximal percentage increase of 550% and 1000% in nucleus accumbens and 250% and 520% in the caudate, respectively.^66^ The median dose of ketamine used in rodent studies was 30 mg kg^−1^ and the lowest dose was 5 mg kg^−1^. Thus, whilst ketamine acts on the dopamine system, it is probably not as potent as these stimulants, although direct comparisons are needed to test this.

In addition it should be noted that *in vitro* studies show ketamine is a DAT antagonist, and thus blockade of dopamine reuptake could contribute to ketamine’s dopaminergic effects. However, its affinity for the DAT (Ki=66.8±25.9 μm) is over an order of magnitude lower than its affinity for the NMDA receptor (Ki=3.1 μm).^67, 68^ It has been observed that the density of dopamine transporter (DAT) in the prefrontal cortex is much lower than in the striatum,^69^ and DAT blockade is not particularly effective in increasing dopamine levels in the prefrontal cortex.^70^ Our meta-analyses indicate there is greater dopamine increase with ketamine in the prefrontal cortex relative to the striatum. This suggests that ketamine’s effects on dopaminergic function are unlikely to be attributable to blockade of dopamine uptake at sub-anaesthetic doses as if that were the case effects would be much larger in the striatum than the prefrontal cortex. However, studies are needed in DAT knock-out mice to investigate whether the effects of ketamine on dopaminergic function are independent of DAT blockade (Box 1).

### Comparison with other NMDA receptor antagonists

In the rodent brain the magnitude of dopamine changes is lower with ketamine relative to those seen with other NMDA receptor antagonists such as PCP and dizocilpine (MK-801), but not memantine. For example, a 5 mg kg^−1^ acute dose of PCP produces a 380–500% increase in prefontal cortical dopamine levels and a 120–190% in nucleus accumbens,^71, 72^ whilst 5–10 mg kg^−1^ doses of ketamine result in increases ~50% in these regions (Table 2). Similarly MK-801 at a dose of 0.1 mg kg^−1^ increased dopamine levels by 190% in PFC and 75% in NAc, respectively.^73^ Moreover, acute PCP at 1 mg kg^−1^ i.v produced a maximal increase of 500% in VTA A10 dopamine neuronal firing rate.^74^ In contrast, acute administration of 20 mg kg^−1^ of the moderate-affinity NMDA receptor antagonist memantine did not change dopamine levels in prefrontal cortex.^75^ Thus the rank order of dopaminergic effects of these NMDA antagonists appears to correspond to the rank order of their NMDA receptor affinities, but there have yet to be direct comparisons. Interestingly, it was shown that dopamine transmission in corticolimbic system was temporally dissociated from PCP-induced locomotor effects.^76^ However, the relationship between ketamine’s effects on dopamine release and the antidepressant, addictive and psychotomimetic effects of ketamine have not been investigated. This is an important future direction to aid understanding of the contribution of dopaminergic mechanisms to these effects (Box 1).

### Comparison between rodent and primate studies

In contrast to the rodent studies, only one of the four primate studies we identified reported a significant change in dopamine release following ketamine administration. Human PET imaging studies, where change in radiotracer binding is used to index dopamine release,^49^ also show inconsistency with some but not all studies showing changes in radiotracer binding with ketamine.^47, 77, 78^ One potential explanation for the discrepancy between rodent and primate studies could be the difference in the timings in which dopaminergic outcome was measured following ketamine injection. In the majority of rodent studies dopaminergic outcome was measured 10–60 min post ketamine injection, whereas in three out of four studies in non-human primates dopaminergic outcome was measured 2 h following ketamine administration. Interestingly, the study which documented 30% increase from baseline measured dopamine levels 45 min post ketamine administration.^37^ Thus the timing of measures in some of the primate studies may have missed the peak dopamine effects, and doses were lower than many of the rodent studies. Further studies in this time range and with higher doses are needed to fully determine the effects of ketamine on the dopamine system in primates.

### Implications for human use of ketamine

Whilst there is a clear need for more studies in primates addressing the issues discussed above, our findings in rodents that acute ketamine causes increases in dopamine levels suggests that ketamine has similar dopaminergic effects as stimulants and a number of other recreational drugs.^79, 80, 81, 82, 83^ This implies that its dopaminergic effects may contribute to ketamine’s abuse potential and the development of dependence.

This also has implications for the use of ketamine in the treatment of depression. Major depression is associated with blunted dopaminergic function.^84, 85^ It has been theorised that depressed individuals cannot obtain reward from normal social interaction because of dopamine deficiency.^86^ Thus, one potential mechanism by which ketamine may be effective in the treatment of depression is by increasing dopamine neuron firing, and consequently dopamine release to enable the appropriate association between social interactions and reward. Supporting this, in a stress-induced rat model of depression, ketamine was shown to restore the decreased dopamine neuron population activity and synaptic plasticity.^28^ However, it should be noted that other mechanisms may also play a role in ketamine’s antidepressant actions, including an action of a metabolite of ketamine on α-amino-3-hydroxy-5-methyl-4-isoxazolepropionic acid receptors.^58^

Sub-anaesthetic doses of ketamine in healthy human subjects acutely induce symptoms comparable to symptoms of schizophrenia^3, 87^ and similar low level symptoms are seen in chronic ketamine abusers.^87, 88^ Moreover, ketamine worsens psychotic symptoms in patients with schizophrenia.^5, 89^ Our findings suggest that ketamine’s psychotomimetic effects may involve the dopaminergic system, consistent with evidence that elevated dopamine synthesis and release capacity are seen in people at risk of developing schizophrenia.^90, 91, 92, 93^ Finally, our findings highlight that it would be useful for future preclinical studies to use methods that can be applied in human studies as well to test these potential implications and aid translation of findings (Box 1).

## Conclusion

Acute ketamine administration leads to increased dopamine levels in the frontal cortex, in the striatum and in the nucleus accumbens in rodents. These findings suggest dopaminergic effects potentially contribute to its acute antidepressant and psychotomimetic effects. Effects are not clear-cut in the primate brain, potentially due to the timing of measures and lower doses used, and there have been few studies of chronic administration in any species. Further studies are required in primates at higher doses and to explore whether the same dopaminergic effects are seen following chronic ketamine administration.

## Figures and Tables

**Table 1:** PET studies of dopaminergic function in non-human primates after ketamine administration compared to control

*Dopamine system studied*	*Study*	*Ketamine*	*Ketamine dose and route of administration*	*State of animal*	*Duration of treatment*	*When the outcome investigated*	*Ligand*	*Radiotracer administration*	*Study design/ control group*	*Analytical method*	*Region of interest*	N^a^	*Outcome measure*	*Change in dopamine measure after ketamine infusion compared to control condition*
Dopamine synthesis	Tsukada *et al.*^36^	Racemic	3 and 10 mg kg^−1 ^h^−1^, i.v	Anaesthetised	Infusion throughout scan	30 mins prior to scan	L-[ β -^11^C]DOPA	i.v	Within/ saline	Graphical analysis (L-[ β -^11^C]DOPA)	Str	4	Dopamine synthesis rate	**↑**
Dopamine release	Hashimoto *et al.*, 2017^38^	R and S-ketamine	0.5 mg kg^−1^	Sub-anaesthetised	Infusion 40 mins	After the end of infusion	[^11^C]raclopride	i.v	Saline	Reference tissue model	Caudate/putamen	4	Δ D2/3 receptor binding potential^b^	**↓**29% (S-Ketamine)
														↔ No change (R Ketamine)
	Tsukada *et al.*^36^	Racemic	3 and 10 mg kg^−1 ^h^−1^, i.v	Anaesthetised	Infusion throughout scan	30 mins prior to scan	[^11^C]raclopride	i.v	Within/ saline	Kinetic analysis ([^11^C]raclopride)	Str	4	Δ Binding potential^b^	**↓**
Dopamine transporter	Tsukada *et al.*^36^	Racemic	3 and 10 mg kg^−1 ^h^−1^, i.v	Anaesthetised	Infusion throughout scan	30 mins prior to scan	[^11^C]β-CFT	i.v	Within/ saline	Kinetic analysis ([^11^C]β-CFT)	Str	4	DAT binding potential	**↓**
	Yamamoto *et al.*^34^	Racemic	0.5 & 1.5 mg kg^−1^	Sub-anaesthetised	Infusion 40 mins	After the end of infusion	[^11^C]β-CFT	Bolus i.v	Within/ saline	Reference tissue model	Ct, Str, Midbrain, Thal	5	DAT binding potential	↔ No change
	Harada *et al.*^40^	Racemic	3 mg kg^−1 ^h^−1^	Anaesthetised	Infusion throughout scan	60 mins before tracers	[^11^C]β-CFT & [^11^C]β-CIT-FE	i.v	Within/ saline	Kinetic analysis	Str	3	DAT binding potential	**↓** ([^11^C]β-CFT)
														↔ No change ([^11^C]β-CIT-FE)
	Tsukada *et al.*^39^	Racemic	3 and 10 mg kg^−1^ h^−1^, i.v	Anaesthetised	Infusion throughout scan	60 mins before tracers	[^11^C]β-CFT & [^11^C]β-CIT-FE	i.v	Within/ saline	Kinetic analysis	Str	5	DAT binding potential	**↓** (3 mg kg^−1^)

Abbreviations: Ct, cortex; DA, dopamine; DAT, dopamine transporter; i.m, intra muscular; i.v, intravenous; NA, not available; PET, positron emission tomography; Str, striatum; Thal, thalamus.

a *N* the sample size represents the total number of animals used for the comparison in question.

b Greater reduction in D2/D3 receptor binding potential after ketamine administration indicates greater dopamine release; **↑**significant increase, **↓**significant decrease and ↔no significant change.

**Table 2:** PET studies of D2/D3 receptor availability in healthy humans after ketamine infusion compared to control condition

*Study*	*Ketamine*	*Ketamine dose and route of administration*	*Duration of administration*	*Ligand*	*Radiotracer administration*	*Study design*	*Region of interest*	N^a^	*(Plasma mean±s.e.m.; ng ml^−1^)*	*Result: change in D2/D3 receptor availability after ketamine infusion compared to control condition*^b^
Aalto *et al.*^45^	Racemic	Infusion=0.80 mg kg^−1^^c^	Infusion 15 mins prior to scan till the end of scan	[^11^C]raclopride	Infusion	Control group: baseline and repeat scan Ketamine group: baseline and ketamine administration	Caudate, putamen, Str	8/8	293±29	↔
Aalto *et al.*^44^	Racemic	Infusion 325.5±57.5 ng ml^−1^	Infusion 15 mins prior to scan- till the end of scan	[^11^C]FLB 457	Infusion	Control group: baseline and repeat scan Ketamine group: baseline and ketamine administration	Ct regions, Thal	8/8	325.5±57.5	**↓**Posterior cingulate ct ↔ (other regions)
Breier *et al.*^41^	Racemic	0.12 mg kg^−1^ (bolus) and 0.65 mg kg^−1^ (infusion)/hour=0.88	Bolus 50 mins after tracer and 1 hour infusion	[^11^C]raclopride	Bolus & infusion	Control group: baseline and saline Ketamine group: baseline and ketamine administration	Str	6/9	NA	**↓**(11%)
Kegeles *et al.*^47^	Racemic	0.12 mg kg^−1^ bolus and 0.65 mg kg^−1 ^h^−1^=0.88	Bolus 50 mins after start of scan and 70 mins infusion	[^11^C]raclopride	Bolus & infusion	Control group: baseline and saline Ketamine group: baseline and ketamine administration	Str subregions	5/5	140±53	↔
Kegeles *et al.*^46^	Racemic	0.2 mg kg^−1^ bolus and 0.4 mg kg^−1 ^h^−1^=1.00	Bolus 120 mins after tracer and 4 h infusion	[123I]IBZM	Bolus & constant infusion	Baseline scan and ketamine administration – within subject	Str	8	191±38	↔
Vernaleken *et al.*^94^	S-ketamine	0.097 mg kg^−1^ bolus and 0.25 mg/ml infusion	35 mins before start of scan infusion was started. Infusion was continued for 30 mins	[18F]-fallypride	Bolus & constant infusion	Placebo/ketamine – within subject	Caudate nucleus, putamen, Thal, ITG, dlPFC	10	NA	**↑**(Caudate nucleus) ↔ (other region)
Smith *et al.*^42^	Racemic	0+1.5 mg kg^−1 ^h^−1^=0.50	Infusion over 20 mins	[^11^C]raclopride	Infusion	Baseline scan and ketamine administration – within subject	Str	7	NA	**↓** (14%)
Vollenweider *et al.*^43^	S-ketamine	0.21+0.84/hour=1.47	Bolus over 5 min	[^11^C]raclopride	Bolus	Placebo/ketamine – within subject	Caudate nucleus, putamen and VS	8	NA	**↓** (14%)

Abbreviations: Ct, cortex; dlPFC, dorsolateral prefrontal cortex; ITG, inferior temporal gyrus; i.v, intravenous; Ket, ketamine; NA, not available; Thal, thalamus; VS, ventral striatum.

a *N* the sample size represents the number of subjects per group.

b Greater reduction in D2/D3 receptor binding potential after ketamine administration indicates greater dopamine release; **↑**significant increase, **↓**significant decrease,↔no significant change.

c Average dose given in the study.

## Suggested future research directions

Preclinical research

1. Further studies to determine the effect of chronic administration (>10 days) of ketamine on dopaminergic function.

2. Studies needed in females to investigate whether there are gender differences.

3. Studies needed to investigate whether there are strain differences.

4. Determine the effects of acute and chronic ketamine on dopaminergic function using translational techniques such as micro-positron emission tomography.

5. Studies to investigate the effect of acute and chronic ketamine on dopamine neuron firing and how that relates with measures of dopamine release and dopamine synthesis capacity.

6. Studies to investigate correlations between dopamine release and psychotomimetic or antidepressant effects of ketamine and other NMDA receptor antagonists.

7. Studies are needed in dopamine transporter (DAT) knock-out (KO) mice to investigate whether the effects of ketamine on dopaminergic function are independent of DAT blockade.

8. Investigate effects of various sub-anaestheic doses of ketamine on dopamine synthesis, transporter and release in primates.

Clinical research

1. Investigate effects of dopamine synthesis and transporter availability following acute ketamine administration.

2. Investigate dopamine release following ketamine challenge using agonist ligand such as [11C] PHNO.

3. Studies needed to investigate dopamine synthesis, release and transporter availability in chronic users.
