# Supplementary material for: The effects of ketamine on dopaminergic function: meta-analysis and review of the implications for neuropsychiatric disorders
Source: Mol Psychiatry. 2017 Oct 3;23(1):59–69. doi: 10.1038/mp.2017.190 (PMC5754467; doi:10.1038/mp.2017.190)
Supplement: Supplementary Material [file mp2017190x1.docx]

**Supplementary Material**

**Supplementary Table 1:** Subject characteristics

| **Author** | **Species** | **Strain** | **Sex** | **Weight (Range or Average ± SEM)** | **Age (weeks/years) (Range or Average ± SEM)** |
| --- | --- | --- | --- | --- | --- |
| Lorrain et al. 2003 ^1^ | Rats | SD | male | 300-350 g | n/a |
| Moghaddam et al. 1997^2^ | Rats | n/a | n/a | n/a | n/a |
| Verma and Moghaddam 1996 ^3^ | Rats | SD | male | 250-350 g | adult |
| Usun et al. 2013^4^ | Rats | SD | male | 400 ± 25 g | n/a |
| Imre et al.2015^5^ | Rats | Wistar | male | 200-250 g | adult |
| Littlewood et al.2006^6^ | Rats | SD | male | 307 g | adult |
| Vaisanen et al.2004^7^ | Rats | Wistar | male | 209-224 g | n/a |
| Micheletti et al 1992^8^ | Rats | n/a | male | 250-280 g | adult |
| Lindefors et al 1997^9^ | Rats | SD | male | 270-300 g | n/a |
| El Iskandrani et al 2015^10^ | Rats | SD | male | 270-330 g | n/a |
| Belujon and Grace 2014^11^ | Rats | Wistar-Kyoto | male | 300-350 g | n/a |
| Kamiyama et al., 2011^12^ | Rats | Wistar | male | n/a | n/a |
| Masuzawa et al., 2003^13^ | Rats | Wistar | male | 250-300 g | n/a |
| Li et al., 2015^14^ | Rats | Wistar | male | 160-180 g | n/a |
| French and Ceci., 1990^15^ | Rats | SD | n/a | n/a | n/a |
| McCown et al., 1982^16^ | Rats | SD | male | 300-350 g | n/a |
| Witkin et al., 2016^17^ | Rats | SD & Wistar | male | 250-330g/308-398g | n/a |
| Carboni et al., 1989^18^ | Rats | SD | male | 180-200g | n/a |
| Chatterjee et al. 2012^19^ | Mice | Swiss albino | male | 30-35 g | n/a |
| Irifune et al. 1997^20^ | Mice | ddY mice | male | 37-49 g | n/a |
| Irifune et al., 1991^21^ | Mice | ddY mice | male | 33-55 g | n/a |
| Tan et al. 2012^22^ | Mice | ICR mice | n/a | n/a | n/a |
| Sorce et al. 2010^23^ | Mice | C57BL/6J | male | n/a | 8-10 weeks |
| Ke et al., 2008^24^ | Mice | C57BL/6J | male | n/a | 8-9 weeks |
| Lai et al., 2013^25^ | Mice | FVB | male | n/a | 8 weeks old |
| Yamamoto et al., 2013^26^ | Primate | Macaca mulatta | male | n/a | 7.8±0.8 years old |
| Adams et al., 2002^27^ | Primate | Macaca mulatta | male | n/a | Adult |
| Onoe et al., 1994^28^ | Primate | Macaca mulatta | male | n/a | Young-Adult |
| Tsukada et al., 2000^29^ | Primate | Macaca mulatta | male | 4-6kg | Young-Adult |

**Abbreviation: SD- Sprague-Dawley, ICR- Institute for Cancer Research, FVB- Friend leukemia virus B, ddY- Deutschland, Denken, and Yoken; n/a- not availabl**

**Supplementary Table 2:** The effect of acute ketamine administration on dopamine level findings by brain region

| **ROI** | **Study** | **N ^a^** | **Dose of ketamine**  **(mg/kg)** | **Route of administration of ketamine** | **Timing of outcome relative to ketamine administration** | **Comparison condition** | **In vivo/Ex vivo** | **Method** | **Maximum change in DA level from control condition (%)** |
| --- | --- | --- | --- | --- | --- | --- | --- | --- | --- |
| **Cortex** | Lorrain et al 2003^1^ | 6 | 18 | s.c | n/a | basal levels | In vivo | Microdialysis, HPLC –ECD | 140% |
|  | Vaisanen et al., 2004^7^ | 4 | 30 | i.p | 2 hours | basal levels | In vivo | Microdialysis, HPLC –ECD | 185% |
|  | Chatterjee *et al.* 2012^19^ | 24 | 100 | i.p | 30 mins | vehicle | Ex vivo | HPLC and ECD | 60% |
|  | Irifune *et al.*1997^20^ | 13 | 30 | i.p | 10 mins | saline | Ex vivo | HPLC and ECD | ↔ |
|  | Moghaddam *et al.*1997^2^ | 13 | 30 | i.p | acute | saline | In vivo | Microdialysis, HPLC-ECD | 150% |
|  | Verma and Moghaddam 1996^3^ | 16 | 30 | i.p | 15 mins | saline | In vivo | In vivo microdialysis, HPLC | 150 % |
|  | Lindefors *et al.* 1997^9^ | 18 | 25 | i.p | At 1 hour and at 2 hours | saline | In vivo | Microdialysis, HPLC -ECD | 400% at 60, mins (less at 120 mins) |
|  | Kamiyama et al. 2011^12^ | 10, 13 | 5 and 25 | i.p | Every 20 mins for 2 hours | saline | In vivo | In vivo microdialysis, HPLC | 50% at 60, mins (5mg/kg) |
|  |  |  |  | i.p |  |  | In vivo |  | 100%, at 60 mins (25mg/kg) |
|  | Sorce et al. 2010 ^23^ | 6 | 30 | i.p | Every 30 mins for 270 mins | saline | In vivo | In vivo microdialysis, HPLC- coulometric detection | 150% |
|  | Irifune 1991 ^21^ | 6-10 | 30 | i.p | 10 and 20 mins post ket administration | saline | Ex vivo | HPLC and ECD | ↔ |
|  | Lai 2013^25^ | 5/6 | 10 and 50 | i.p | 30 mins or 2 hours after ket | saline | Ex vivo | HPLC | ↔ |
|  | Witkin et al., 2016^17^ | 11 | 10 | s.c | 40 mins | vehicle | In vivo | In vivo microdialysis, HPLC and ECD | 240% |
| **STR** | Usun et al., 2013^4^ | 7 | 10 and 20 | s.c | Immediately for 60 mins | basal levels | In vivo | Voltammetric analysis | 68%  after 10mg/kg ketamine |
|  |  |  |  | s.c |  |  | In vivo |  | 52.8% after 20mg/kg ketamine |
|  | Irifune *et al.1997^20^* | 13 | 30 | i.p | 10 mins | saline | Ex vivo | HPLC and ECD | ↔ |
|  | Moghaddam *et al.*1997^2^ | 14 | 30 | i.p | acute | saline | In vivo | Microdialysis, HPLC -ECD | 25% |
|  | Verma and Moghaddam 1996^3^ | **7** | 30 | i.p | 20 mins | saline | In vivo | In vivo microdialysis, HPLC | 30% |
|  | Irifune 1991^21^ | 6-10 | 30 | i.p | 10 and 20 mins post ket administration | saline | Ex vivo | HPLC and ECD | ↔ |
|  | Lai 2013^25^ | 5/6 | 10 and 50 | i.p | 30 mins or 2 hours after ket | saline | Ex vivo | HPLC | ↔ |
|  | Carboni et al. 1989^18^ | n/a | 10 | s.c | n/a | saline | In vivo | Microdialysis HPLC and ECD | ↔ |
| **Hp** | Irifune *et al.*199*7^20^* | 13 | 30 | i.p | 10 mins | saline | Ex vivo | HPLC and ECD | ↔ |
|  | Imre *et al.*2005^5^ | 30 | 12 | s.c | 30 mins | saline | Ex vivo | HPLC and ECD | 62.6% |
|  | Irifune 1991^21^ | 6-10 | 30 | i.p | 10 and 20 mins post ket administration | saline | Ex vivo | HPLC and ECD | ↔ |
| **NAcc** | Irifune *et al.*1997^20^ | 13 | 30 | i.p | 10 mins | saline | Ex vivo | HPLC and ECD | ↔ |
|  | Littlewood *et al.*2006^6^ | 4/6 | 10, 25 | s.c | collections for 2 hours | saline | In vivo | Microdialysis, HPLC | 50% for 25mg/kg |
|  |  |  |  | s.c |  |  | In vivo |  | 55% for 10mg/kg |
|  | Irifune 1991^21^ | 6-10 | 30 | i.p | 10 and 20 mins post ket administration | saline | Ex vivo | HPLC and ECD | ↔ |
|  | Masuzawa et al. 2003^13^ | 15 | 50 and 100 | i.p | Every 20 mins for 100 mins | saline | In vivo | In vivo microdialysis, HPLC and ECD | 40% after 50mg/kg ket |
|  |  |  |  | i.p |  |  | In vivo |  | 140% after 100mg/kg |
|  | Witkin et al. 2016^17^ | 8 | 25 | i.p | 60 mins | vehicle | In vivo | In vivo microdialysis, HPLC and ECD | 75% |
| **BS** | Irifune *et al.1997^20^* | 13 | 30 | i.p | 10 mins | saline | Ex vivo | HPLC and ECD | ↔ |
|  | Irifune 1991^21^ | 6-10 | 30 | i.p | 10 and 20 mins post ket administration | saline | Ex vivo | HPLC and ECD | ↔ |
| **VP** | Littlewood *et al.*2006^6^ | 4/6 | 10 and 25 | s.c | collections for 2 hours | saline | In vivo | Microdialysis, HPLC | ↔ |
| **L Hem.** | Li et al., 2015^14^ | 24 | 10, 30, 60 | i.p | 45 mins post ket | saline | Ex vivo | HPLC and ECD | 3%  (10mg/kg) |
|  |  |  |  | i.p |  |  | Ex vivo |  | 11%  (30mg/kg) |
|  |  |  |  | i.p |  |  | Ex vivo |  | 3%  (60mg/kg) |

**Abbreviations:** NAcc- nucleus accumbens, Hp- hippocampus, Str- striatum; VP- ventral pallidum; BS- brainstem; L Hem.- left hemisphere; DA- dopamine; Ket- ketamine; HPLC-ECD-High-performance liquid chromatography electrochemical detection; i.p - intra peritoneal ; s.c – subcutaneous; n/a – not available; significant increase, significant decrease, ↔ no significant change

N ^a^ the sample size represents the total number of animals used for the comparison in question

**Supplementary Table 3:** Dopamine neuron firing in VTA following acute and chronic administration of ketamine in chloral hydrate anaesthetised rats.

| **Author** | **Dose of ket**  **(mg/kg)** | **N ^a^** | **Route of administration** | **Treatment** | **When the outcome investigated** | **Control group** | **Methods used** | **Outcome measure** | **Result (% Difference)** |
| --- | --- | --- | --- | --- | --- | --- | --- | --- | --- |
| French and Ceci 1990, ^15^ | 100µmoles/kg | 8-21 rats | i.v | acute | n/a | Basal levels | In vivo electrophysiology | Firing rate | 40% |
| El Iskandrani et al., 2015^10^ | 10mg/kg | 81 neurons, 10 rats | i.p | acute | 30 minutes post ket | saline | In vivo electrophysiology | Average firing rate | ↔ |
|  |  |  |  |  |  |  |  | burst activity | ↔ |
|  |  |  |  |  |  |  |  | population activity | 113% |
| El Iskandrani et al., 2015^10^ | 10mg/kg | 190 neurons, 13 rats | i.p | Chronic (3 days once per day) | On Day 3, 30 minutes following last drug injection. | saline | In vivo electrophysiology | Average firing rate | ↔ |
|  |  |  |  |  |  |  |  | burst activity | ↔ |
|  |  |  |  |  |  |  |  | population activity | ↔ |
| Belujon and Grace 2014^11^ | 5mg/kg | 16 rats, 93 neurons | i.p | acute | 20 mins, 2 hours or 24 hours after ket | Naïve controls | Extracellular recordings | Population activity | 180% at 2 hours compared to 20 mins |
|  |  |  |  |  |  |  |  | basal firing rate | 36.8% at 20 mins compared to home cage animals |
|  |  |  |  |  |  |  |  | proportion of action potentials in burst | 81.8% in 20 mins compared to home cage animals |
| Witkin et al., 2016^17^ | 3, 10 and 17 mg/kg | 20-28 rats | i.v | acute | 10mins post ket | Vehicle-treated | In vivo electrophysiology | No of spontaneously active cells | 77% (3mg/kg)  62% (10mg/kg)  139% (17mg/kg) |
|  |  |  |  |  |  |  |  | Firing rate | ↔ |
|  |  |  |  |  |  |  |  | % of Spikes in Bursts | ↔ |

**Abbreviations:** DA- dopamine; Ket- ketamine; i.p - intra peritoneal; i.v- intravenous; significant increase, significant decrease, ↔ no significant change

N ^a^ the sample size represents the total number of animals used for the comparison in question

**Supplementary Table 4:** Dopamine levels following chronic administration of ketamine

| **ROI** | **Study** | **N ^a^** | **Dose and route of administration of ketamine**  **(mg/kg)** | **Duration of treatment** | **Timing of outcome** | **Control condition** | **Method** | **Result (% Diff in DA levels)** |
| --- | --- | --- | --- | --- | --- | --- | --- | --- |
| **Ct** | Chatterjee *et al.* 2012^19^ | 48 | 100 i.p | 10 days | On day 11 | saline | HPLC-ECD | 88% |
|  | Tan et al., 2012^22^ | 12 | 30 i.p | Daily for 3 months | Not clear | saline | Immunoassay | 184% |
|  | Lindefors et al., 1997^9^ | 18 | 25 i.p | Once daily for 8 days | On day 8 | saline basal levels | Microdialysis HPLC-ECD | 138% |
| **Str** | Chatterjee *et al.* 2012^19^ | 48 | 100 i.p | 10 days | On day 11 | saline | HPLC-ECD | 130% |
|  | Tan et al., 2012^22^ | 12 | 30 i.p | Daily for 3 months | Not clear | saline | DA Research ELISA | ↔ |
|  | Micheletti *et al.* 1992^8^ | 17 | 15 per day in liquid diet | For 50 days | On day 50 | controls | Reverse phase chromatography with ECD | ↔ |
| **Hp** | Chatterjee *et al.* 2012^19^ | 48 | 100 i.p | 10 days | On day 11 | saline | HPLC-ECD | ↔ |
| **MB** | Tan et al., 2012^22^ | 12 | 30 i.p | Daily for 3 months | Not clear | saline | Immunoassay | ↔ |
| **Cb** | Tan et al., 2012^22^ | 12 | 30 i.p | Daily for 3 months | Not clear | saline | Immunoassay | ↔ |
| **L Hem** | Li et al., 2015^14^ | 24 | 10, 30 and 60mg/kg i.p | For 1, 2 and 3 weeks | At the end of 1, 2 and 3 weeks | saline | HPLC and ECD | 32% |

**Abbreviations:** Ct- cortex; Str- striatum; Hp- hippocampus; MB- midbrain; Cb- Cerebellum; L.Hem-left hemisphere; DA- dopamine; Ket- ketamine; HPLC-ECD-High-performance liquid chromatography electrochemical detection; i.p - intra peritoneal; significant increase, significant decrease, ↔ no significant change

N ^a^ the sample size represents the total number of animals used for the comparison in question

**Supplementary Table 5** – Dopamine levels following administration of anaesthetic doses of ketamine (>100mg/kg) in rodents

| **ROI** | **Study** | **N ^a^** | **Dose of ketamine** **(mg/kg)** | **Route of administration of ketamine** | **Timing of outcome relative to ketamine administration** | **Comparison condition** | **In vivo**  **/Ex vivo** | **Method** | **Maximum change in dopamine levels from control condition (%)** |
| --- | --- | --- | --- | --- | --- | --- | --- | --- | --- |
| **Ct** | Irifune *et al*., 1991^21^ | 6-10 | 150 | i.p | 120 mins | saline | Ex-vivo | HPLC and ECD | ↔ |
|  | Irifune *et al.*1997^20^ | 13 | 150 | i.p | 10 and 120 mins | saline | Ex vivo | HPLC and ECD | ↔ |
|  | Ke et al., 2008^24^ | 16 | 350 (7 consecutive doses of 50) | i.p | 2 weeks | saline | Ex vivo | HPLC and ECD | ↔ |
| **Str** | McCown *et al*., 1982^16^ | 10 | 150 | i.p | 30 mins | saline | Ex-vivo | HPLC | ↔ |
|  | Irifune *et al*., 1991^30^ | 6-10 | 150 | i.p | 120 mins | saline | Ex-vivo | HPLC and ECD | ↔ |
|  | Irifune *et al.*1997^20^ | 13 | 150 | i.p | 10 and 120 mins | saline | Ex vivo | HPLC and ECD | ↔ |
|  | Ke et al., 2008^24^ | 16 | 350 (7 consecutive doses of 50) | i.p | 2 weeks | saline | Ex vivo | HPLC and ECD | ↔ |
| **Hp** | Irifune *et al*., 1991^30^ | 6-10 | 150 | i.p | 120 mins | saline | Ex-vivo | HPLC and ECD | ↔ |
|  | Irifune *et al.*1997^20^ | 13 | 150 | i.p | 10 and 120 mins | saline | Ex vivo | HPLC and ECD | ↔ |
| **NAcc** | Irifune *et al*., 1991^30^ | 6-10 | 150 | i.p | 120 mins | saline | Ex-vivo | HPLC and ECD | ↔ |
|  | Irifune *et al.*1997^20^ | 13 | 150 | i.p | 10 and 120 mins | saline | Ex vivo | HPLC and ECD | ↔ |
|  | Ke et al., 2008^24^ | 16 | 350 (7 consecutive doses of 50) | i.p | 2 weeks | saline | Ex vivo | HPLC and ECD | ↔ |
| **BS** | Irifune *et al*, 1991^30^ | 6-10 | 150 | i.p | 120 mins | saline | Ex-vivo | HPLC and ECD | ↔ |
|  | Irifune *et al.*1997^20^ | 13 | 150 | i.p | 10 and 120 mins | saline | Ex vivo | HPLC and ECD | ↔ |

**Abbreviations:** Ct- cortex; Str- striatum; Hp- hippocampus; NAcc- nucleus accumbens; BS- brainstem; HPLC-ECD-High-performance liquid chromatography electrochemical detection; i.p - intra peritoneal; ↔ No change

N ^a^ the sample size represents the total number of animals used for the comparison in question

**Supplementary Table 6** – Acute and chronic studies of dopamine levels in non-human primate studies.

| **ROI** | **Study** | **N ^a^** | **Dose and route of administration of ketamine**  **(mg/kg)** | **Duration of treatment** | **When the outcome investigated** | **Study design/ Control comparison** | **Method** | **DA release/levels** | |
| --- | --- | --- | --- | --- | --- | --- | --- | --- | --- |
|  |  |  |  |  |  |  |  | **Outcome measure** | **Result (% Diff)** |
| **Cortex** | Yamamoto et al., 2013^26^ | 3 | 1.5 i.v | Acute: Infusion over 3 hours | 3 hours after start of infusion | Within subject/ saline | Microdialysis, reverse-phase- HPLC-ECD | DA levels (% baseline) | ↔ |
| **Str** | Adams et al., 2002^27^ | 2 (8 events) | 5 i.m | Acute, i.m | 45 min after injection | Within subject/ from baseline | MRI-directed in vivo microdialysis, HPLC-ECD | DA (% baseline) | 30% over baseline |
|  | Onoe et al., 1994^28^ | 3 | 5 i.m | Acute, i.m | 2hr30mins after injection | Within subject/ from baseline | microdialysis and HPLC | Dopamine release | ↔ |
|  | Tsukada et al., 2000^29^ | 4 | 3 and 10 mg/kg/hr infusion posterior tibia vein cannula | Acute: Infusion over 2 hours | 120mins after start of infusion | Within subject/saline | Microdialysis and HPLC | Dopamine release | ↔ |

**Abbreviations:** Ct- cortex; Str- striatum; DA- dopamine; Ket- ketamine; HPLC-ECD-High-performance liquid chromatography electrochemical detection, i.m - intra muscular; i.v – intravenous; ↑ significant increase, ↓ significant decrease, ↔ no significant change

N ^a^ the sample size represents the total number of animals used for the comparison in question

Note that in non-human primate studies the dose of 3 mg/kg used is sufficient to cause anaesthesia. 1.5mg/kg is interpreted as sub-anaesthetic dose.

**Supplementary figure 1: Flow chart of the inclusion of studies for the meta-analysis on dopaminergic function following acute ketamine treatment.**

Records identified through database searching
(n = 1263)

## Eligibility

## Included

## Screening

## Identification

Records after duplicates removed
(n = 839)

Records screened
(n = 839)

Studies included in qualitative synthesis
(total studies: n = 25)

21-rodent

4-primate

Full-text articles excluded from meta-analysis because there were fewer than 5 studies

1) Dopamine outcomes in primate brain (n=4)

2) Dopaminergic outcomes after chronic ketamine administration (n = 3)

3) Ketamine effects on dopamine neuron firing (n=2)

4) Ketamine effects on dopamine in the hippocampus (n=1)

Full-text articles excluded for the following reasons
(n = 40)

No extractable dopamine data (n=11)

*In vitro* studies and imaging studies (n = 26)

No dopamine measures (n= 3)

Records excluded
(n = 774)

Studies included in meta-analysis
(total studies: n = 15)

Dopamine levels in rodent

Cortex: n=11

Striatum: n=6

Nucleus Accumbens: n=5

Full-text articles assessed for eligibility
(n = 65)

**Supplementary Figure 2:** Funnel plots of dopamine levels in frontal cortex for all studies

**Funnel plots of standard error by standardized mean difference**

**
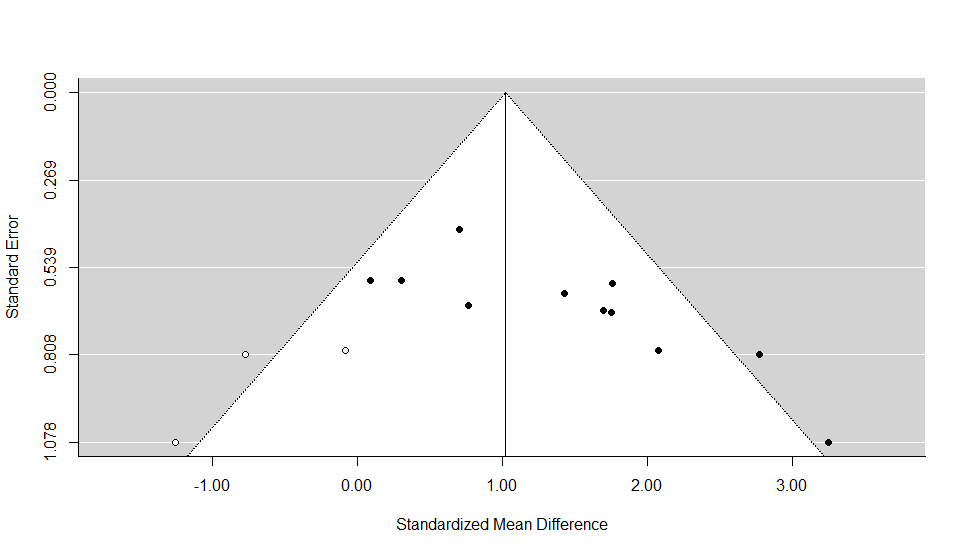
**

1. Lorrain DS, Baccei CS, Bristow LJ, Anderson JJ, Varney MA. Effects of ketamine and N-methyl-D-aspartate on glutamate and dopamine release in the rat prefrontal cortex: modulation by a group II selective metabotropic glutamate receptor agonist LY379268. *Neuroscience* 2003; **117**(3)**:** 697-706.

2. Moghaddam B, Adams B, Verma A, Daly D. Activation of glutamatergic neurotransmission by ketamine: a novel step in the pathway from NMDA receptor blockade to dopaminergic and cognitive disruptions associated with the prefrontal cortex. *The Journal of neuroscience : the official journal of the Society for Neuroscience* 1997; **17**(8)**:** 2921-2927.

3. Verma A, Moghaddam B. NMDA receptor antagonists impair prefrontal cortex function as assessed via spatial delayed alternation performance in rats: Modulation by dopamine. Jan 1996. *The Journal of Neuroscience* 1996; **.16**(1)**:** pp.

4. Usun Y, Eybrard S, Meyer F, Louilot A. Ketamine increases striatal dopamine release and hyperlocomotion in adult rats after postnatal functional blockade of the prefrontal cortex. *Behavioural Brain Research* 2013; **256:** 229-237.

5. Imre G, Salomons A, Jongsma M, Fokkema DS, Den Boer JA, Horst GJT. Effects of the mGluR2/3 agonist LY379268 on ketamine-evoked behaviours and neurochemical changes in the dentate gyrus of the rat. *Pharmacology, Biochemistry and Behavior* 2006; **.84**(3)**:** pp.

6. Littlewood CL, Jones N, O'Neill MJ, Mitchell SN, Tricklebank M, Williams SC. Mapping the central effects of ketamine in the rat using pharmacological MRI. *Psychopharmacology* 2006; **186**(1)**:** 64-81.

7. Vaisanen J, Ihalainen J, Tanila H, Castren E. Effects of NMDA-receptor antagonist treatment on c-fos expression in rat brain areas implicated in schizophrenia. *Cellular and molecular neurobiology* 2004; **24**(6)**:** 769-780.

8. Micheletti G, Lannes B, Haby C, Borrelli E, Kempf E, Warter JM *et al.* Chronic administration of NMDA antagonists induces D2 receptor synthesis in rat striatum. *Brain research Molecular brain research* 1992; **14**(4)**:** 363-368.

9. Lindefors N, Barati S, OConnor WT. Differential effects of single and repeated ketamine administration on dopamine, serotonin and GABA transmission in rat medial prefrontal cortex. *Brain Research* 1997; **759**(2)**:** 205-212.

10. El Iskandrani KS, Oosterhof CA, El Mansari M, Blier P. Impact of subanesthetic doses of ketamine on AMPA-mediated responses in rats: An in vivo electrophysiological study on monoaminergic and glutamatergic neurons. *Journal of Psychopharmacology* 2015; **.29**(7)**:** pp.

11. Belujon P, Grace AA. Restoring mood balance in depression: Ketamine reverses deficit in dopamine-dependent synaptic plasticity. *Biological Psychiatry* 2014; **.76**(12)**:** pp.

12. Kamiyama H, Matsumoto M, Otani S, Kimura SI, Shimamura KI, Ishikawa S *et al.* Mechanisms underlying ketamine-induced synaptic depression in rat hippocampus-medial prefrontal cortex pathway. *Neuroscience* 2011; **177:** 159-169.

13. Masuzawa M, Nakao S, Miyamoto E, Yamada M, Murao K, Nishi K *et al.* Pentobarbital inhibits ketamine-induced dopamine release in the rat nucleus accumbens: a microdialysis study. *Anesthesia and analgesia* 2003; **96**(1)**:** 148-152, table of contents.

14. Li B, Liu ML, Wu XP, Jia J, Cao J, Wei ZW *et al.* Effects of ketamine exposure on dopamine concentrations and dopamine type 2 receptor mRNA expression in rat brain tissue. *International journal of clinical and experimental medicine* 2015; **8**(7)**:** 11181-11187.

15. French ED, Ceci A. Non-competitive N-methyl-D-aspartate antagonists are potent activators of ventral tegmental A10 dopamine neurons. *Neuroscience letters* 1990; **119**(2)**:** 159-162.

16. McCown TJ, Mueller RA, Breese GR. Effects of anesthetics and electrical stimulation on nigrostriatal dopaminergic neurons. *The Journal of pharmacology and experimental therapeutics* 1983; **224**(3)**:** 489-493.

17. Witkin JM, Monn JA, Schoepp DD, Li X, Overshiner C, Mitchell SN *et al.* The Rapidly Acting Antidepressant Ketamine and the mGlu2/3 Receptor Antagonist LY341495 Rapidly Engage Dopaminergic Mood Circuits. *The Journal of pharmacology and experimental therapeutics* 2016; **358**(1)**:** 71-82.

18. Carboni E, Imperato A, Perezzani L, Di Chiara G. Amphetamine, cocaine, phencyclidine and nomifensine increase extracellular dopamine concentrations preferentially in the nucleus accumbens of freely moving rats. *Neuroscience* 1989; **28**(3)**:** 653-661.

19. Chatterjee M, Verma R, Ganguly S, Palit G. Neurochemical and molecular characterization of ketamine-induced experimental psychosis model in mice. *Neuropharmacology* 2012; **.63**(6)**:** pp.

20. Irifune M, Fukuda T, Nomoto M, Sato T, Kamata Y, Nishikawa T *et al.* Effects of ketamine on dopamine metabolism during anesthesia in discrete brain regions in mice: comparison with the effects during the recovery and subanesthetic phases. *Brain research* 1997; **763**(2)**:** 281-284.

21. Irifune M, Shimizu T, Nomoto M. Ketamine-induced hyperlocomotion associated with alteration of presynaptic components of dopamine neurons in the nucleus accumbens of mice. Oct 1991. *Pharmacology, Biochemistry and Behavior* 1991; **.40**(2)**:** pp.

22. Tan S, Lam WP, Wai MSM, Yu WHA, Yew DT. Chronic Ketamine Administration Modulates Midbrain Dopamine System in Mice. *PloS one* 2012; **7**(8).

23. Sorce S, Schiavone S, Tucci P, Colaianna M, Jaquet V, Cuomo V *et al.* The NADPH Oxidase NOX2 Controls Glutamate Release: A Novel Mechanism Involved in Psychosis-Like Ketamine Responses. *Journal of Neuroscience* 2010; **30**(34)**:** 11317-11325.

24. Ke JJ, Chen HI, Jen CJ, Kuo YM, Cherng CG, Tsai YPN *et al.* Mutual enhancement of central neurotoxicity induced by ketamine followed by methamphetamine. *Toxicology and applied pharmacology* 2008; **227**(2)**:** 239-247.

25. Lai CC, Lee LJ, Yin HS. Combinational effects of ketamine and amphetamine on behaviors and neurotransmitter systems of mice. *Neurotoxicology* 2013; **37:** 136-143.

26. Yamamoto S, Ohba H, Nishiyama S, Harada N, Kakiuchi T, Tsukada H *et al.* Subanesthetic doses of ketamine transiently decrease serotonin transporter activity: A PET study in conscious monkeys. *Neuropsychopharmacology* 2013; **.38**(13)**:** pp.

27. Adams BW, Bradberry CW, Moghaddam B. NMDA antagonist effects on striatal dopamine release: microdialysis studies in awake monkeys. *Synapse* 2002; **43**(1)**:** 12-18.

28. Onoe H, Inoue O, Suzuki K, Tsukada H, Itoh T, Mataga N *et al.* Ketamine Increases the Striatal N-[C-11]Methylspiperone Binding in-Vivo - Positron Emission Tomography Study Using Conscious Rhesus-Monkey. *Brain Research* 1994; **663**(2)**:** 191-198.

29. Tsukada H, Harada N, Nishiyama S, Ohba H, Sato K, Fukumoto D *et al.* Ketamine decreased striatal [(11)C]raclopride binding with no alterations in static dopamine concentrations in the striatal extracellular fluid in the monkey brain: multiparametric PET studies combined with microdialysis analysis. *Synapse* 2000; **37**(2)**:** 95-103.

30. Irifune M, Shimizu T, Nomoto M. Ketamine-induced hyperlocomotion associated with alteration of presynaptic components of dopamine neurons in the nucleus accumbens of mice. *Pharmacology, biochemistry, and behavior* 1991; **40**(2)**:** 399-407.
